# Ultrahigh omnidirectional, broadband, and polarization-independent optical absorption over the visible wavelengths by effective dispersion engineering

**DOI:** 10.1038/s41598-019-46413-3

**Published:** 2019-07-08

**Authors:** Yeonghoon Jin, Junghoon Park, Yoonhyuk Rah, Jaeho Shim, Kyoungsik Yu

**Affiliations:** School of Electrical Engineering, Korea Advanced Institute of Science and Technology (KAIST), 291 Daehak-Ro, Yuseong-Gu, Daejeon 34141 Republic of Korea

**Keywords:** Nanocavities, Electrical and electronic engineering

## Abstract

Achieving perfect light absorption at a subwavelength-scale thickness has various advantageous in terms of cost, flexibility, weight, and performance for many different applications. However, obtaining perfect absorbers covering a wide range of wavelengths regardless of incident angle and input polarization without a complicated patterning process while maintaining a small thickness remains a challenge. In this paper, we demonstrate flat, lithography-free, ultrahigh omnidirectional, polarization-independent, broadband absorbers through effective dispersion engineering. The proposed absorbers show day-integrated solar energy absorption up to 96%, which is 32% better than with lossy semiconductor/metal absorbers. The proposed simple yet effective method can be applied to light absorption thin film structures based on various types of highly lossy semiconductor materials, including emerging 2D materials.

## Introduction

Perfect light absorption has attracted much attention due to its versatile usage for energy harvesting^1–3^, photodetection^4^, coloring^5^, bio-sensing^6^, radiative cooling^7^ and thermal emission^8^. Among these, broadband and wide-angle light absorption in the visible wavelength range is especially suited to solar energy harvesting, photodetection and coloring applications. Achieving such perfect absorption at subwavelength-scale thickness has various advantages in terms of cost, weight, flexibility, and electrical performance as compared to using conventional bulk structures^9^. Researchers have used a variety of techniques to provide substantial light absorption over a wide range of wavelengths, regardless of incident-angle and input polarization. Efficient light absorption has been achieved using high-index nanostructures^10,11^, photonics crystals^12,13^, high-impedance meta-materials^14^, and metallic nano-structures^1^. However, such techniques typically include complicated wavelength-scale patterning processes and still exhibit incident-angle or polarization-dependent absorption properties.

On the other hand, a new type of anti-reflection (AR) coating using highly absorbing semiconductor films on metal substrates (e.g., ~25 nm amorphous germanium (a-Ge) layer on Au substrate) can achieve lithography-free, incident-angle and polarization-independent absorption^15^. Unlike conventional AR coatings that typically require quarter-wave-thick dielectric layers with a refractive index of geometric mean of the substrate and air^16^, the semiconductor-metal two-layer structure achieves destructive interference from a lossy semiconductor layer much thinner than the usual quarter-wave thickness^15^. Unlike an ideal mirror or a perfect electric mirror, of which the phase shift at the reflection surface is limited to π only, typical metal layers with finite conductivity allow penetration of the incident electromagnetic waves. The penetration depth depends on the refractive indices of the materials on the metal layer and thus provide additional phase shift when compared to the perfect electric mirror. Accordingly, the amount of phase shift upon reflection at such a high index semiconductor-metal interface can be greater than π, allowing destructive interference with a thinner layer. Such non-conventional phase shift has been interpreted as a perfect magnetic mirror, which shows a phase shift of 0 (or 2π) upon reflection at the mirror interface. By using ultrathin Si_3_N_4_ and Ge films on an Au substrate, a lossy magnetic mirror has been demonstrated^17^. In addition, this AR coating method is applicable to flexible substrates with a high degree of roughness^18^. The choice of the lossy top-layer material for this two-layer optical absorption structure can be quite versatile as long as its extinction coefficient is larger than 0.64 (ultrathin case)^19^. Examples include amorphous silicon, gallium arsenide^20^ and transition metal dichalcogenides (TMDCs)^21,22^.

Park *et al*.^20^ revealed that the Brewster mode of the Ge-on-metal structure is responsible for omnidirectional absorption up to 60° at the resonance wavelength. However, more complete and broadband absorption is desirable because this scheme shows high absorption only around the resonance wavelength. Moreover, because the Brewster mode mostly exists in transvers magnetic (TM) polarization only, the absorption of transverse electric (TE) polarization is much lower than that of TM polarization. Some research groups have addressed these problems by using a spacer below the highly absorbing layer^23–25^, metal-semiconductor-metal cavity structure^26^, metamaterials^27–29^, lossy magnetic mirrors^17^, and cavity tuning for aesthetic purpose^30,31^. However, so far, it remains a challenge to make incident-angle and polarization-independent optical absorbers that cover a wide range of wavelengths (over the whole visible range) without using complex fabrication procedures. Metal-insulator multilayer absorbers also have been well investigated, but in these cases most of light is absorbed in the metal layers, which is difficult to contribute to charge carriers^32,33^.

In this paper, we experimentally demonstrate flat, lithography-free, omnidirectional (>93% absorption even at the incident angle of 60°), broadband absorbers through effective dispersion engineering. Firstly, we analyse absorption and reflection from ultrathin lossy semiconductor on metal substrate, and reveal that such two-layer absorbers support both radiative and non-radiative modes. In addition, we clearly show distinct limitations of the two-layer absorbers in achieving broadband omnidirectional perfect absorptions. To overcome such problems, we derive theoretical conditions for ideal perfect absorption, and mimic such ideal conditions by engineering the effective dispersion of three thin film layers (dielectric-semiconductor-metal layers). We also show that this simple yet effective technique can be applicable to other highly lossy semiconductors, such as TMDCs.

## Results and Discussion

### Two-layer semiconductor absorbers

**Figure 1:**
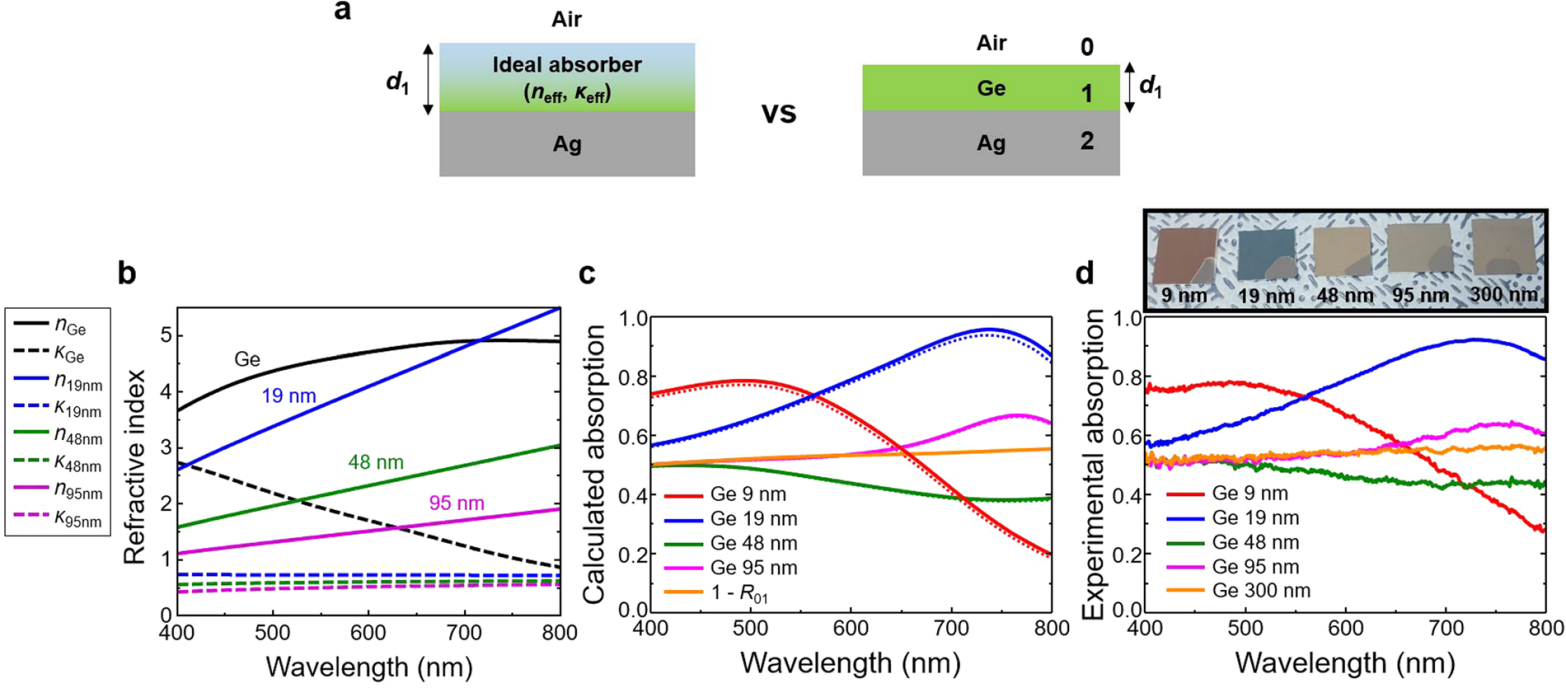
(**a**) Schematic of comparison between an ideal absorber and a semiconductor (Ge) absorber on Ag substrates. (**b**) Complex refractive indices of the Ge and the ideal absorbers with different thickness (*d*_1_ = 19, 48, and 95 nm). The solid and dashed lines represent the real and imaginary parts of the refractive index. (**c**) Calculated and (**d**) Experimental absorption spectra of the semiconductor absorbers with different thickness (*d*_1_ = 9, 19, 48, 95, and 300 nm). The solid and dotted lines in (**c**) represent the total (Ge + Ag) and the exclusive (Ge only) absorption, respectively. Inset in (**d**) shows fabricated samples.

We first imagine an ideal two-layer absorber with a fictitious absorption material deposited on a silver (Ag) substrate (Fig. 1a). Ag was chosen as the metal back reflector because of its low absorption losses for the wavelength range of our interest (400–800 nm). (see Supplementary Information Fig. S1 for comparison with other metals). Reflection coefficient of the two-layer system can be expressed as

1 $$r=\frac{{r}_{01}+{r}_{12}{e}^{2i{\beta }_{1}{d}_{1}}}{1+{r}_{01}{r}_{12}{e}^{2i{\beta }_{1}{d}_{1}}}$$

where *r*_mn_ = (*p*_m_ − *p*_n_)/(*p*_m_ + *p*_n_) is the reflection coefficient at the interface between the *m*^th^ and *n*^th^ layer, and *p*_m_ = *n*_m_cos(*θ*_m_) for TE polarization and *p*_m_ = *n*_m_/cos(*θ*_m_) for TM polarization. Here, *n*_m_ is the complex refractive index of the *m*^th^ layer, the propagation angle *θ*_m_ = sin^−1^ (sin(*θ*_0_)/*n*_m_) is obtained by Snell’s law, *θ*_0_ is the incident-angle from the air (*m* = 0), *β*_1_ is the longitudinal wavenumber inside the 1^st^ layer, and *d*_1_ is the thickness of the 1^st^ layer. For perfect absorption (*r* = 0 in Eq. (1)), the refractive index (*n*_eff_, *κ*_eff_) of the fictitious material (ideal absorber) needs to vary with the input wavelength and also depends on its thickness. Figure 1b shows several examples when the layer thicknesses (*d*_1_) are 19, 48, and 95 nm (blue, olive and magenta lines, respectively). The solid and dashed lines represent the real and imaginary parts of the refractive index. In reality, such an ideal absorber does not exist. However, if a material (or a combination of multiple layers) closely follows the dispersion properties of an ideal absorber, broadband perfect light absorption can be expected. Before expanding our discussion into the multiple-layer absorption system, we first choose germanium (Ge) and compare its dispersion with the ideal absorber (Fig. 1a,b). The refractive indices of the Ge (Fig. 1b, black lines) and the ideal absorber are different each other, and we can predict that the Ge is far from the ideal absorber. Refractive indices of the Ge and the ideal absorber are very close only around the wavelength of 720 nm when *d*_1_ is 19 nm (black and blue line).

Even though the Ge cannot be such an ideal absorber, it is worth looking at some physical fundamentals of the semiconductor/metal absorber to understand its limitations. We first investigated the Ge/Ag absorber with the Ge layer thickness of 9, 19, 48, 95, or 300 nm. Figure 1c shows the calculated absorption (absorptance) at normal incidence from 400 to 800 nm using a transfer matrix method (TMM)^34^. The solid lines represent the total absorption by the Ge and Ag layers, while the dotted lines show the absorption only by the Ge layer, excluding the absorption by the Ag layer. As we predicted from Fig. 1b, the Ge layer 19 nm (blue line) shows maximum absorption (95.7%) around the wavelength of 740 nm. When it is thicker than 19 nm, the absorption is decreased. In order to obtain near-unity absorption or near-zero reflection, as a rule of thumb, the first partially reflected wave from the interface between air and the lossy semiconductor (Ge) layer should destructively interfere with other subsequently reflected waves (multiple-reflection within the Ge layer). However, when the Ge layer becomes as thick as or thicker than 95 nm, most of the light propagating in the Ge layer is absorbed within one round-trip (two times of the Ge thickness), and there remains little light that can contribute to destructive interference with the first partially reflected wave (Supplementary Information Fig. S2). Accordingly, the absorption from a thicker Ge layer (for example, 300 nm-thick Ge layer, orange line in Fig. 1d) is almost same as 1 − *R*_01_ (orange line in Fig. 1c), where *R*_01_ is the reflectance from the air and the Ge interface, such that *R*_01_ = |(1 − *n*_Ge_)/(1 + *n*_Ge_)|^2^ where *n*_Ge_ is the complex refractive index of Ge. Figure 1d shows the experimental absorption spectra that match well with the calculations, and the inset (black border line) represents a photograph of the fabricated samples showing various colours depending on the Ge layer thickness.

The Ge 19 nm absorber showed the absorption of 95.7%, which is much thinner (~*λ*/8*n*_Ge_) than conventional quarter-wave-thick AR coatings. It is originated from the non-trivial phase shift between the Ge layer and the Ag layer. To explain it, we introduced two conditions. Perfect absorption can be obtained when the numerator of Eq. (1) becomes zero, and this occurs when the two terms in the numerator have the same magnitude (|*r*_01_| = |*r*_12_exp(2*iβ*_1_*d*_1_)|) and their phase difference is π. The magnitude- and phase-matching conditions for the two-layer perfect absorbers are thus given by Δ*R* = 0 and *Ψ*_Diff_ = π, where

2 $${\rm{\Delta }}R={R}_{01}-{R}_{12}{\rm{e}}{\rm{x}}{\rm{p}}(\,-\alpha {d}_{1})$$

3 $${\Psi }_{{\rm{Diff}}}=-\,{\Psi }_{01}+{\Psi }_{12}+2{\Psi }_{{\rm{Ge}}\cdot }$$

*R*_*mn*_ = |*r*_*mn*_|^2^, *α* = 4π*κ*_1_/*λ*, *κ*_1_ is the extinction coefficient of the Ge layer, and *Ψ*_01_ and *Ψ*_12_ are the reflection phase shift from air to the Ge layer and the Ge layer to the Ag layer, respectively (*Ψ*_01_ = arg(*r*_01_), *Ψ*_12_ = arg(*r*_12_)). Here, *Ψ*_Ge_ corresponds to the propagating phase shift within the Ge layer, and *Ψ*_Diff_ is the phase difference between the two terms in the numerator of Eq. (1). Although *Ψ*_Diff_ can be in general (2*p* + 1)π where *p* is an integer, we are mainly interested in the smallest order (*p* = 0) to achieve subwavelength-scale layer thickness. Assume that the magnitude-matching condition (Δ*R* = 0) is almost satisfied. In terms of the phase-matching condition (*Ψ*_Diff_ = π), *Ψ*_01_ is almost constant at π due to the high index contrast between air and Ge (Supplementary Information Fig. S3a), thus *Ψ*_12_ + 2*Ψ*_Ge_ $\simeq$ 2π. If the Ag layer were replaced by a perfect electric conductor (*Ψ*_12_ = π), *Ψ*_Ge_ would become π/2, which means that the Ge layer thickness should be *λ*/4*n*_Ge_ to achieve *Ψ*_Diff_ = π. However, when *Ψ*_12_ is larger than π, *Ψ*_Ge_ can be smaller than π/2 resulting in thinner than *λ*/4*n*_Ge_. Because the field-penetration depth into the Ag layer increases with *n*_Ge_ (Fig. S3b), *Ψ*_12_ can be as high as 330° and the corresponding Ge layer thickness can thus be thinner than *λ*/4*n*_Ge_. Such non-conventional phase shift upon reflection at the Ge/Ag interface also can be understood as a magnetic mirror^17^.

**Figure 2:**
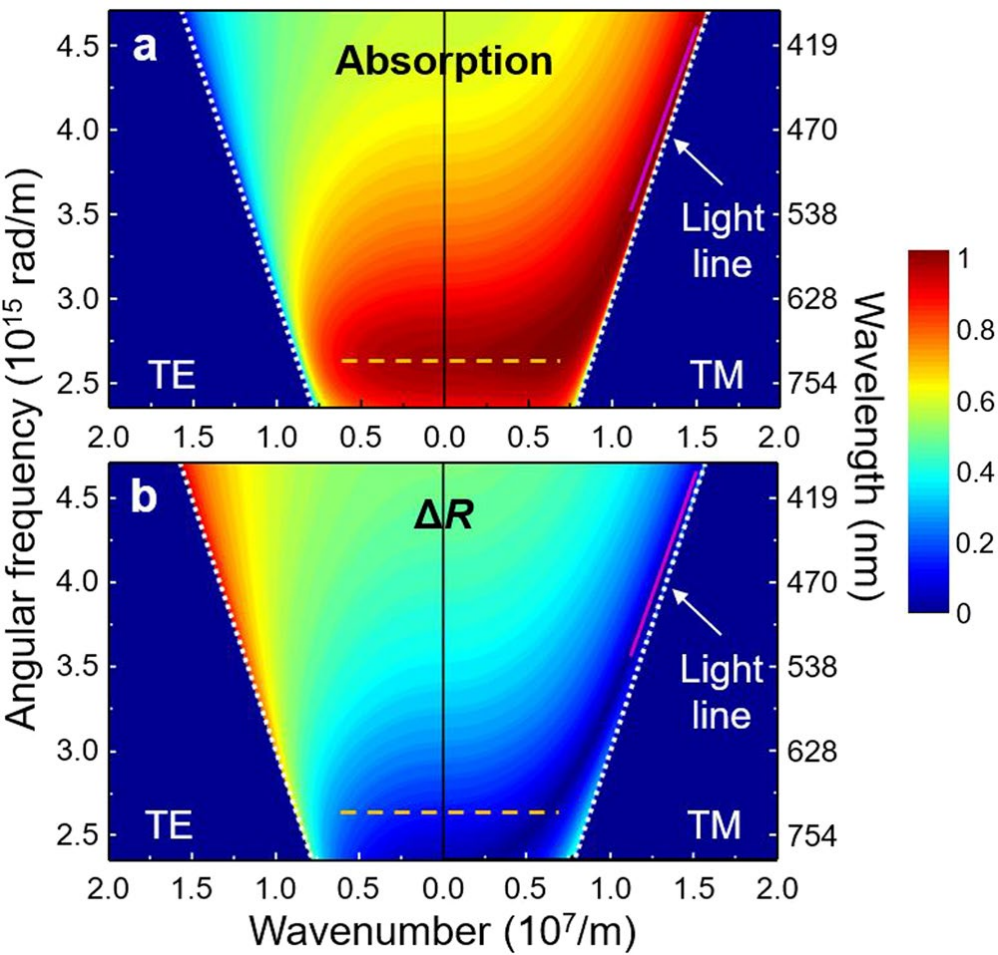
Dispersion relationship of (**a**) Absorption and (**b**) Magnitude difference (Δ*R*) for the Ge 19 nm/Ag case of TE (left panel) and TM (right panel) polarizations. The white dotted lines represent the light line in air. The flat dispersion region (orange dashed line) and high slope region (magenta solid line) are marked.

To investigate incident-angle and input-polarization dependency, we show the dispersion relationship for the absorption (1 − |*r*|^2^) and the magnitude difference (Δ*R*, Eq. 2) of the Ge 19 nm/Ag absorber using the TMM in Fig. 2. The horizontal axis represents the surface-parallel component of the wavevector, *β* (m^−1^), and the vertical axis represents the angular frequency, *ω* (rad/m), corresponding to the visible wavelength. The left and right panels show TE and TM polarization, respectively, and the white dotted lines are the light line in air. Strong absorption supported by the Brewster mode is observed in Fig. 2a and it matches well with a previous report^20^. By further analysis, we revealed that the high absorption regions following the Brewster mode can be separated into two regions that show physically different phenomena: a high slope region around the Brewster angle (magenta solid line) and a flat dispersion region (orange dashed line).

First, in the high slope region, the magnitude-matching condition (Δ*R* ~ 0 as indicated in Fig. 2b) plays more important roles in achieving perfect absorption than does the phase-matching condition (Supplementary Information Fig. S4). This region is located around the Brewster angle (*θ*_B_ = arctan(*n*_Ge_/*n*_air_)), which is around 80° for TM polarization due to the high refractive index contrast. Note that there exists no such high slope region for TE polarization (left panel) because the Brewster angle exists only for TM polarization. Since there is almost no reflected light at the air/Ge interface at the Brewster angle (*R*_01_ $\simeq$ 0), *R*_12_exp(−*αd*_1_) is also close to zero according to Eq. (2). This region can be understood as a non-radiative mode^35^, where there is no any reflected light and thus the phase matching is no longer meaningful. In addition, the high slope region *almost* always exists regardless of the Ge layer thickness (see Supplementary Information Fig. S5). As a result, the high slope region is considered as a non-radiative mode and only exists only around the Brewster angle.

Second, the flat dispersion region showing omnidirectional absorption exists for both TE and TM polarizations up to 60° around the resonance wavelength (around 720 nm). The maximum absorption in this region can be as high as 99.9%. In this region, Δ*R* (Fig. 2a) shows low values and *Ψ*_Diff_ (Fig. S4) is close to π, satisfying both matching conditions of Eqs (2) and (3). In this flat dispersion region, unlike in the high slope region where *R*_01_ = 0 (no interference due to no reflected light), the destructive interference (both Δ*R* and *Ψ*_Diff_ are important) among the partially reflected waves becomes critical to suppress the first partially reflected light, *R*_01_. Accordingly, the flat dispersion region is radiative mode, and requires destructive interference to achieve anti-reflection. The destructive interference phenomenon is strongly affected by the Ge layer thickness. Because the thick Ge layer significantly attenuates the partially reflected waves and leaves little light to participate the destructive interference (Fig. S2), this flat dispersion region only appears when the Ge layer is sufficiently thin. For example, as shown in Fig. S5, there is no flat dispersion region in the Ge 300 nm/Ag case.

In addition, we would like to emphasize that the large index contrast between the air and the Ge layer is responsible for this flat dispersion property^36^. A vertical component of the wavevector (longitudinal direction) inside Ge shows little change even if the incident-angle (*θ*_air_) is significantly varied, resulting in the incident angle-independent property of *Ψ*_12_ and *Ψ*_Ge_ (Fig. S4). In this case, *Ψ*_Diff_ is also insensitive to the incident-angle, showing omnidirectional absorption. It is worth noting that the high slope region (non-radiative mode) always exists regardless of the Ge layer thickness (Fig. S5), whereas the flat dispersion region (radiative mode) exists only when the Ge layer is thin. Therefore, both radiative and non-radiative modes can exist at the same time only when the Ge layer is thin enough. The coexistence of both absorption regions (the flat dispersion region and the high slope region) is a unique characteristic of an ultrathin lossy Gires-Tournois interferometer. However, at the same time, due to the properties of the radiative mode, high absorption in a wide range of wavelengths is prohibited for this two-layer Ge absorber.

### Designing ideal absorbers through effective dispersion engineering

**Figure 3:**
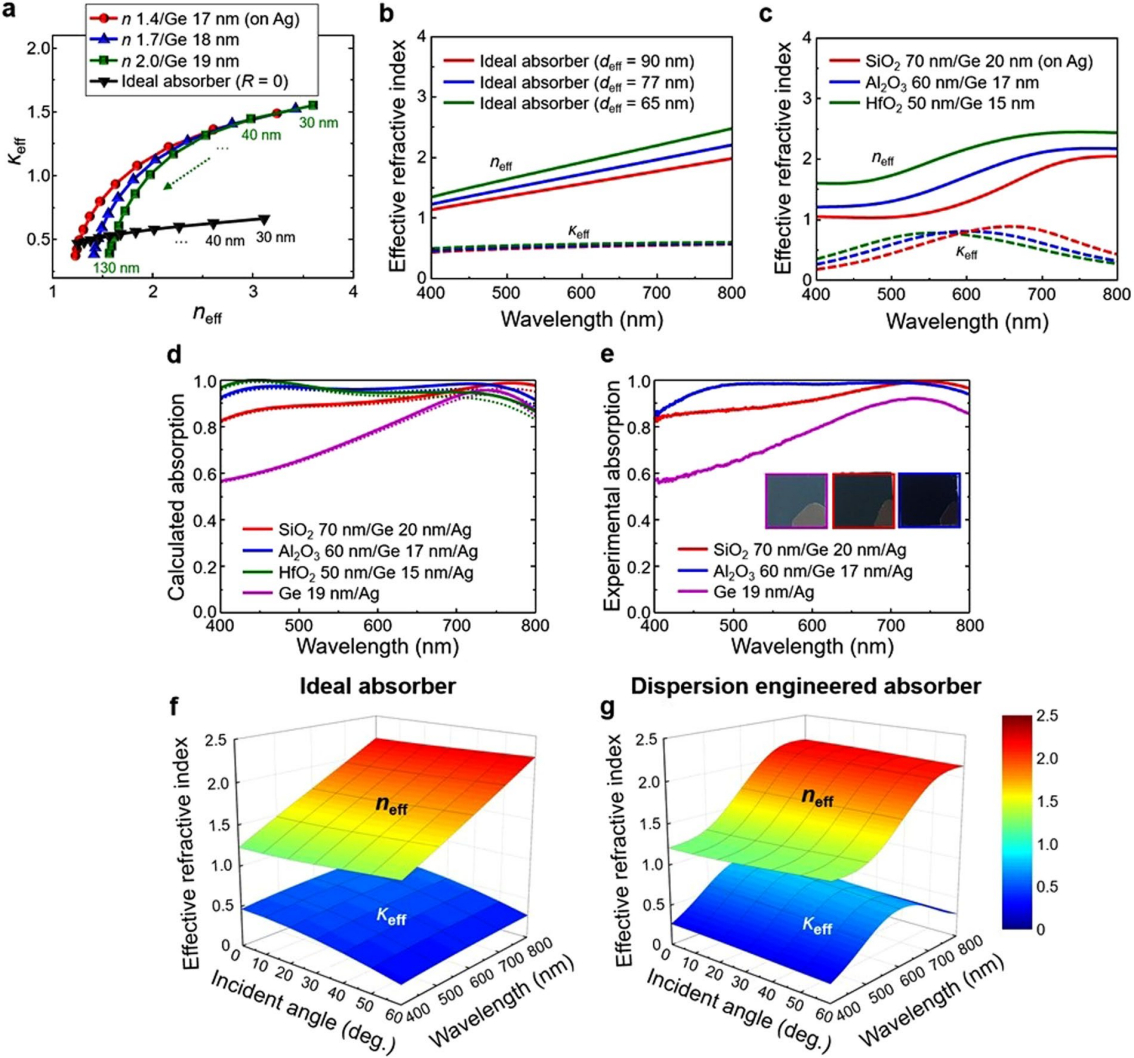
(**a**) Effective refractive index (*n*_eff_, *κ*_eff_) of the ideal absorber (black inverted triangle) and the effective dispersion engineered absorbers on the Ag substrates depending on the thickness of both absorbers (30–130 nm, intervals of 10 nm) at the wavelength of 600 nm. The effective refractive index of (**b**) the ideal absorber and (**c**) the effective dispersion engineered absorbers with *d*_eff_ of 90, 77, and 65 nm. The solid and dashed lines represent the real and imaginary parts of the refractive index. (**d**) Calculated and (**e**) Experimental absorption spectra of the effective dispersion engineered absorbers. The solid and dotted lines represent the total absorption (Ge + Ag layers) and the absorption only by the Ge layer. Insets show photographs of the fabricated samples. The effective refractive index of (**f**) the ideal absorber (*d*_eff_ = 77 nm) and (**g**) the effective dispersion engineered absorber (Al_2_O_3_ 60 nm/Ge 17 nm) depending on the incident angle (0–60°) and the wavelength (400–800 nm). The upper side is a real part (*n*_eff_) and the lower side is an imaginary part (*κ*_eff_).

As shown in Fig. 1b, to get the ideal absorber in a real case, both real and imaginary parts of the refractive index should be reduced as compared with the Ge. It means that lossless and low-index materials should be added to the Ge to get the ideal absorber. Accordingly, we tried to mimic an ideal absorber by introducing lossless and low-index mediums on the Ge, which can be understood as an effective dispersion engineering. We first calculated refractive index of the ideal absorber at the wavelength of 600 nm when the thickness is increased from 30 to 130 nm with 10 nm intervals (Fig. 3a, black inverted triangle). It was obtained by numerically solving Eq. (1) equals to zero, where the 2^nd^ layer (substrate) is Ag. By adding different index lossless materials (*n* = 1.4, 1.7, and 2.0) on top of the Ge layer, we also calculated the effective refractive index with different effective thickness (30–130 nm) (see Supplementary Information Fig. S6 for the effective refractive index calculation). For example, for the *n* = 2.0/Ge 19 nm (green square) case, when the effective thickness (*d*_eff_) is 30 nm, the thickness of the *n* = 2.0 layer is 11 nm. The effective index can be controlled by changing the effective thickness (*d*_eff_) and the index of the additional medium. We can see that black inverted triangles (ideal absorber) and the others are crossed at some specific thicknesses. It means that a lossless medium on top of the Ge configuration can mimic the ideal absorber. For example, the effective index of the *n* = 1.7/Ge 18 nm (blue triangle) with *d*_eff_ of 110 nm is nearly matched with that of the ideal absorber (black inverted triangle) with *d*_eff_ of 110 nm.

Guided by the above results, we carefully designed absorbers to mimic the ideal absorber by using low-index and lossless oxide materials on the Ge layer. The refractive indices for the ideal absorbers (*d*_eff_ = 90, 77 and 65 nm) are shown in Fig. 3b. The solid and dashed lines represent the real and imaginary parts of the refractive index. The effective refractive indices of the dispersion engineered absorbers (SiO_2_ 70 nm/Ge 20 nm, Al_2_O_3_ 60 nm/Ge 17 nm and HfO_2_ 50 nm/Ge 15 nm) on the Ag substrates are also shown in Fig. 3c. The effective thicknesses (*d*_eff_) of them are 90, 77 and 65 nm. We emphasize that both Fig. 3b,c are similar to each other, which means that the ideal absorber can be imitated in a real case by controlling the material’s effective dispersion. By using the effective indices from Fig. 3c, we calculated absorption of them (Fig. 3d). The absorption of each absorbers is increased as compared with the two-layer Ge 19 nm/Ag absorber (magenta line). The solid lines represent the total absorption and the dotted lines show the absorption only by the Ge layer. The experimental absorption spectra shown in Fig. 3e are in good agreement with the calculations, and the insets show photographs of fabricated samples (Ge 19 nm/Ag (magenta border line), SiO_2_ 70 nm/Ge 20 nm/Ag (red border line) and Al_2_O_3_ 60 nm/Ge 17 nm/Ag (blue border line)). The absorption of the overall engineered absorbers shows as high as 99%.

**Figure 4:**
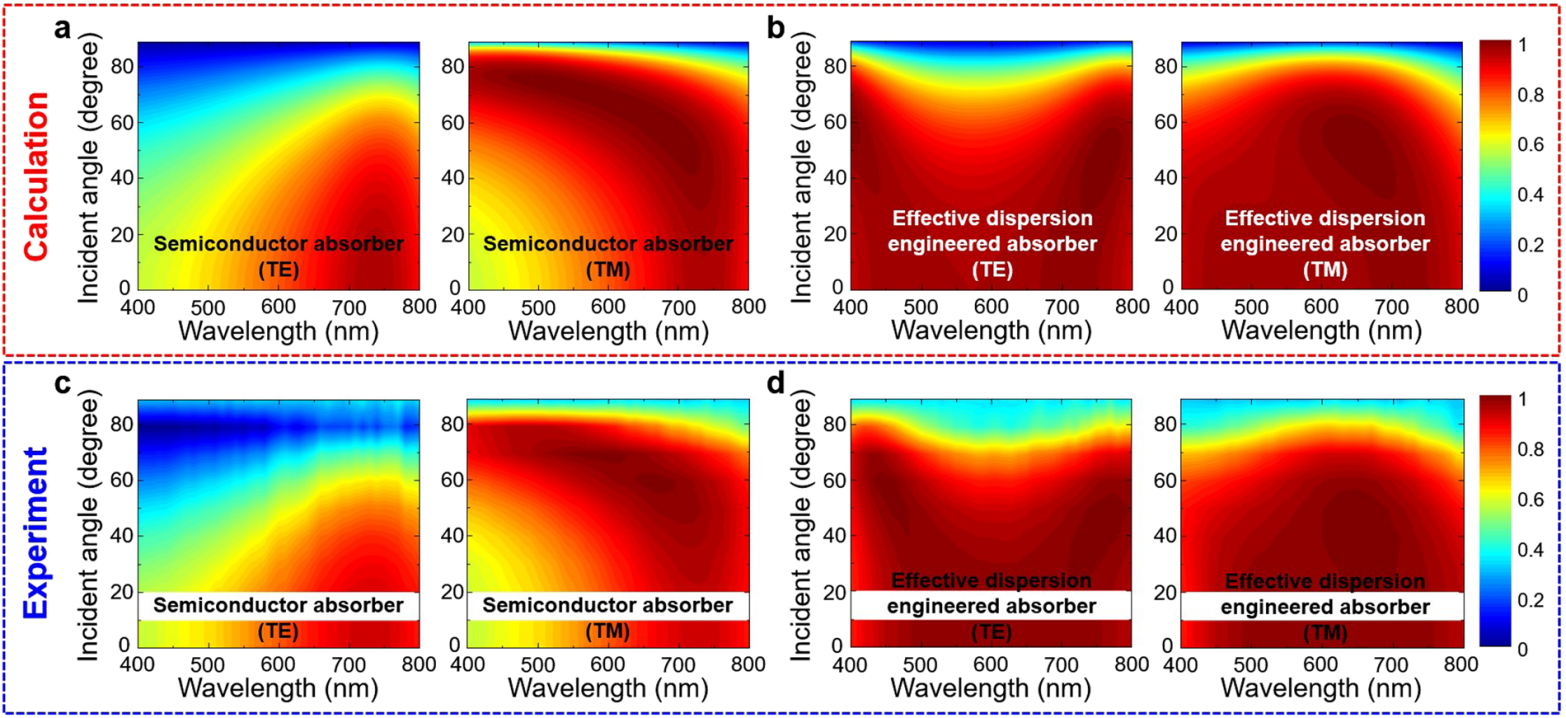
Absorption dispersion depending on the wavelength (400 to 800 nm) and incident-angle (0 to 90°). The calculated absorption 2D contour maps of (**a**) Ge 19 nm/Ag and (**b**) Al_2_O_3_ 60 nm/Ge 17 nm/Ag (effective dispersion engineered absorber) case. The left and right panels of each part represent TE and TM polarization. The experimental absorption maps of (**c**) Ge 19 nm/Ag and (**d**) Al_2_O_3_ 60 nm/Ge 17 nm/Ag are shown.

For further investigation of the incident angle and input polarization dependency, we show the effective index of the ideal absorber (*d*_eff_ = 77 nm) and the effective dispersion engineered absorber (Al_2_O_3_ 60 nm/Ge 17 nm) on the Ag substrate depending on the incident-angle (0–60°) and the wavelength (400–800 nm) for TE polarization (Fig. 3f,g). The upper side is a real part (*n*_eff_) and the lower side is an imaginary part (*κ*_eff_). The effective indices of the ideal absorber and the effective dispersion engineered absorber are very similar to each other. It is worth noting that our proposed dispersion engineering method can be used not only for normal incidence but also for oblique incidence for both TE and TM polarization. To visualize intuitively the angle-dependent absorption properties, we used two-dimensional (2D) contour maps, in which the horizontal and vertical axes represent the input wavelength (400–800 nm) and the incident-angle (0–90°), respectively. In Fig. 4, the calculated and experimentally measured absorption of the basic semiconductor/metal absorber (Ge 19 nm/Ag, Fig. 4a,c) are compared with the effective dispersion engineered absorber (Al_2_O_3_ 60 nm/Ge 17 nm/Ag, Fig. 4b,d) for both polarizations. Our dispersion engineered absorber shows highly omnidirectional and broadband absorption for both polarizations. The experimentally measured absorption values (Fig. 4c,d) are in good agreement with the calculated results (Fig. 4a,b). The experimental data for the incident angle of 10° is missing owing to the limitations of the instrument used in our measurements.

At a glance, our effective dispersion engineered absorber seems like conventional AR coatings, because of the configuration (an oxide layer on top of the Ge layer). However, our absorbers are fundamentally different from those of conventional AR coatings^37^. For example, the thickness of such conventional AR coatings is *λ*/4 while the thickness of our absorber is almost *λ*/8 (Al_2_O_3_ 60 nm, *n* = 1.67, wavelength of 800 nm). Moreover, our absorbers show highly omnidirectional and broadband (the whole visible range) absorption independent of polarizations.

### Quantitative analysis for solar energy absorption applications

**Figure 5:**
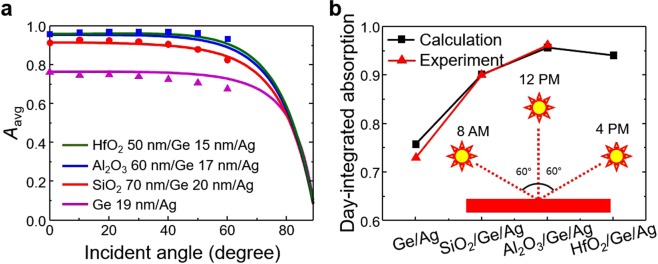
(**a**) Integrated solar energy absorption spectra (Eq. 4) of the two-layer and three-layer optical absorbers. The solid lines and the scatter symbols represent calculated and experimental absorption, respectively. (**b**) Day-integrated solar energy absorption of the proposed absorbers with the calculated (black square) and the experimental (red triangle) absorption. The inset shows a schematic of the day-integrated solar energy absorption scenario.

For more quantitative analysis in solar energy absorption applications, we introduced integrated solar energy absorption, *A*_*avg*_*(θ)*, which averages the total absorption of the AM1.5 solar irradiance spectrum ranging from 400 to 800 nm at the incident-angle of *θ*. It is given by

4 $${A}_{avg}(\theta )=\frac{{\int }_{400}^{800}I(\theta ,\lambda )\cos (\theta )A(\theta ,\lambda )d\lambda }{{\int }_{400}^{800}I(\theta ,\lambda )\cos (\theta )d\lambda }$$

where *I*(*θ*,*λ*) is the AM1.5 solar irradiance, and *A*(*θ*,*λ*) is the absorption at the input wavelength *λ* and the incident-angle *θ*. The integrated absorption calculated using Eq. (4) is shown in Fig. 5a, and the x-axis represents the incident-angle from 0° to 90°. The solid lines represent the calculated absorption and the scatter symbols represent the experimental absorption values from 0° to 60° at intervals of 10°. The experimental absorption for the Al_2_O_3_ 60 nm/Ge 17 nm/Ag case (blue square) is higher than 93% even at 60°, whereas the Ge 19 nm/Ag case (magenta triangle) results in only 67% at the same incident-angle. In addition, we also introduced day-integrated solar energy absorption^38^ (Fig. 5b), which integrates the total average absorption of the AM1.5 solar irradiance spectrum from 400 to 800 nm with the incident-angle variation between −60° and 60° (corresponding to approximated solar movement from 8 AM to 4 PM, moving 15° in 1 h). The inset in Fig. 5b is an illustration of the day-integrated solar energy absorption. The calculated day-integrated solar energy absorption values are plotted as black squares. The experimental absorption (red triangle) in the Ge 19 nm/Ag case is 73%, in the SiO_2_ 70 nm/Ge 20 nm/Ag case is 90%, and in the Al_2_O_3_ 60 nm/Ge 17 nm/Ag case is 96%, which is an increase of up to 32% compared with the two-layer case (Ge 19 nm/Ag).

### Applicability to other highly lossy semiconductors

**Figure 6:**
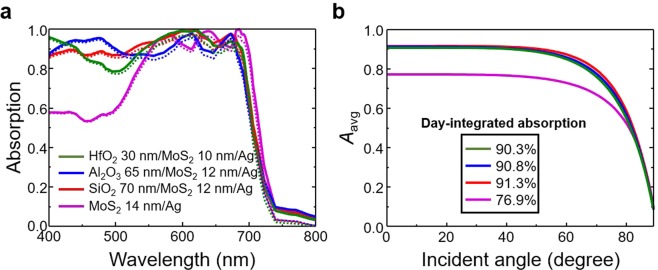
Optical properties of the MoS_2_ absorbers. (**a**) Calculated absorption spectra of the two-layer and three-layer absorbers. The solid and dotted lines represent the total absorption (MoS_2_ + Ag layers) and the exclusive absorption (MoS_2_ layer only), respectively. (**b**) Integrated solar energy absorption (Eq. 4) of the MoS_2_ absorbers. The inset values show the day-integrated solar energy absorption.

Our effective dispersion engineering method can be applicable to other highly lossy semiconductors, including 2D TMDC materials. As an example, we show the calculated absorption of the effective dispersion engineered molybdenum disulfide (MoS_2_) absorbers in Fig. 6a. The solid lines represent the total absorption and the dotted lines represent the absorption only by the MoS_2_ layer. The overall absorption spectra are enhanced (HfO_2_ 30 nm/MoS_2_ 10 nm/Ag (olive line), Al_2_O_3_ 65 nm/MoS_2_ 12 nm/Ag (blue line), and SiO_2_ 70 nm/MoS_2_ 12 nm/Ag (red line)) when compared to the optimized basic semiconductor absorber (MoS_2_ 14 nm/Ag, magenta line). They show better absorption performance, even with the thinner MoS_2_ layer (14 nm in magenta line versus 10 nm in olive line). We also calculated the integrated solar energy absorption (Eq. 4) with respect to the incident-angle at wavelengths ranging from 400 to 700 nm (Fig. 6b). They maintained 90% absorption up to the incident-angle of 50°. In addition, the day-integrated solar energy absorption values are shown on the black border line. The SiO_2_ 70 nm/MoS_2_ 12 nm/Ag (red line) case shows 91.3%: much better than the MoS_2_ 14 nm/Ag case of 76.9%. Therefore, this simple but robust method clearly can be applied to various highly lossy semiconductors.

## Conclusion

In summary, we revealed that the high absorption conditions exhibited by lossy semiconductor/metal two-layer optical absorbers can be explained by two physically distinct phenomena: the radiative mode and the non-radiative mode. The radiative mode (flat dispersion region) appears only when the semiconductor layer is sufficiently thin to facilitate destructive interference. The non-radiative mode (high slope region) is not related to the interference phenomena and is almost independent of the semiconductor layer thickness if the semiconductor layer can absorb all the incident light (at least 10 nm in our Ge absorber case). As a result, both modes can coexist at the same time if the lossy semiconductor layer is sufficiently thin. However, due to the properties of the radiative mode, high absorption in a wide range of wavelengths is prohibited for this two-layer semiconductor absorber.

We introduced a fictitious material (ideal absorber) that doesn’t exist in nature and calculated conditions to obtain such an ideal absorber. To mimic the ideal absorber in a real case, we engineered effective dispersion of a semiconductor (Ge) by introducing lossless and low-index mediums. By carefully designing, we are able to significantly enhance light absorption over a wide range of input wavelengths and incident-angles for both TE and TM polarization. They showed day-integrated solar energy effective absorption up to 96%. In addition, our effective dispersion engineering method can be applied to other lossy semiconductor such as 2D TMDC materials. We expect that our proposed method to achieve polarization-independent, wide-angle, broadband, and lithography-free optical absorbers can find a variety of applications in optoelectronics and energy harvesting.

## Methods

### Sample preparation

The Ag layer was deposited on the Si substrate by an electron-beam evaporator at a pressure of ~10^−6^ torr and a rate of 1Å/s, and Ge was subsequently deposited at the same conditions. Before deposition, the native oxide layer on the Si substrate was removed by buffered oxide etchant, and then it was rinsed by di-ionized water, and dehydrated on a 120 °C hot plate. The Ag layer thickness was chosen to be 150 nm, which is much thicker than the typical skin depth for the wavelength range of our interest. The SiO_2_ and Al_2_O_3_ layers were deposited on the Ge layer by a radio-frequency sputter system at a pressure of ~10^−6^ torr.

### Optical measurements

The normal incidence reflection was measured by a combination of a halogen lamp and a spectrometer (Dongwoo Optron, Monora 500i). The obtained reflection values are normalized by a reference dielectric mirror (ThorLabs) with high reflectivity (>99%) in the wavelength range from 400 nm to 800 nm. The amount of absorption can be obtained from the normalized reflection values. The angle and wavelength-dependent absorption spectra (Fig. 4c,d) were obtained by an ellipsometer (Woollam, V-VASE) with a reflection mode. The incident-angles were varied from 20° to 90°. The absorption of the unpolarized light (Fig. 5a,b) was measured by a UV-visible spectrometer (Perkin Elmer, Lambda 1050) and the incident-angles were varied from 0° to 60°.

### Theoretical calculation

The complex refractive indices of Ag and Ge were experimentally measured using a spectroscopic ellipsometer (Woollam M2000D), and shown in the Supplementary Information Fig. S7. The complex permittivity values for MoS_2_^39^ as well as the refractive indices of SiO_2_, Al_2_O_3_ and HfO_2_ were taken from the literature^40–42^. The TMM^34^ was used for theoretical absorption and reflection calculation. The absorption was calculated from the normalized reflection, ignoring light transmission through the thick Ag layer (150 nm). The effective refractive index calculation is explained in Fig. S6. The absorption of the unpolarized light (Fig. 5a,b) was calculated by averaging the absorption of TE and TM polarization.

## Supplementary information

Ultrahigh omnidirectional, broadband, and polarization-independent optical absorption over the visible wavelengths by effective dispersion engineering
