# Supplementary material for: Ultrahigh omnidirectional, broadband, and polarization-independent optical absorption over the visible wavelengths by effective dispersion engineering
Source: Sci Rep. 2019 Jul 8;9:9866. doi: 10.1038/s41598-019-46413-3 (PMC6614405; doi:10.1038/s41598-019-46413-3)
Supplement: Supplementary file 1 — Ultrahigh omnidirectional, broadband, and polarization-independent optical absorption over the visible wavelengths by effective dispersion engineering [file 41598_2019_46413_MOESM1_ESM.pdf]

Supplementary Information

Ultrahigh omnidirectional, broadband, and polarization-independent optical absorption over the visible wavelengths by effective dispersion engineering

Yeonghoon Jin, Junghoon Park, Yoonhyuk Rah, Jaeho Shim and Kyoungsik Yu*

School of Electrical Engineering, Korea Advanced Institute of Science and Technology (KAIST), 291 Daehak-Ro, Yuseong-Gu, Daejeon 34141, Republic of Korea

E-mail: ksyu@kaist.edu

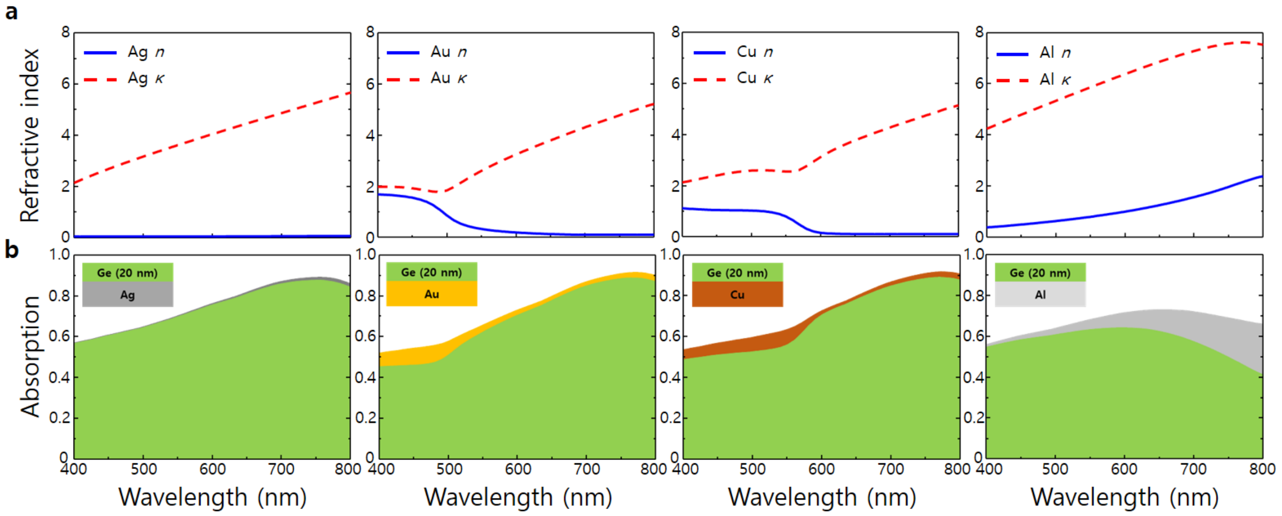

Figure S1. (a) Complex refractive indices of various metals (Ag, Au, Cu and Al) and (b) Calculated absorption of the two-layer optical absorbers with different metal substrates. The amount of absorption in each layer is filled with different colors.

Figure S1 shows complex refractive indices[1] of various metals (silver (Ag), gold (Au), copper (Cu) and aluminium (Al)) and the calculated absorption of the Ge layer on metal layers. The absorption spectra by the Ge layer are filled with green color, while that of the metal layers are filled with different colors. As shown in Figure S1b, the Ag layer shows the lowest parasitic losses compared with the other metal layers. Since absorption from metals increases when real (n) and imaginary parts (κ) of the refractive index become comparable, Ag is a good candidate for the back reflector in the visible range (400 – 800 nm).

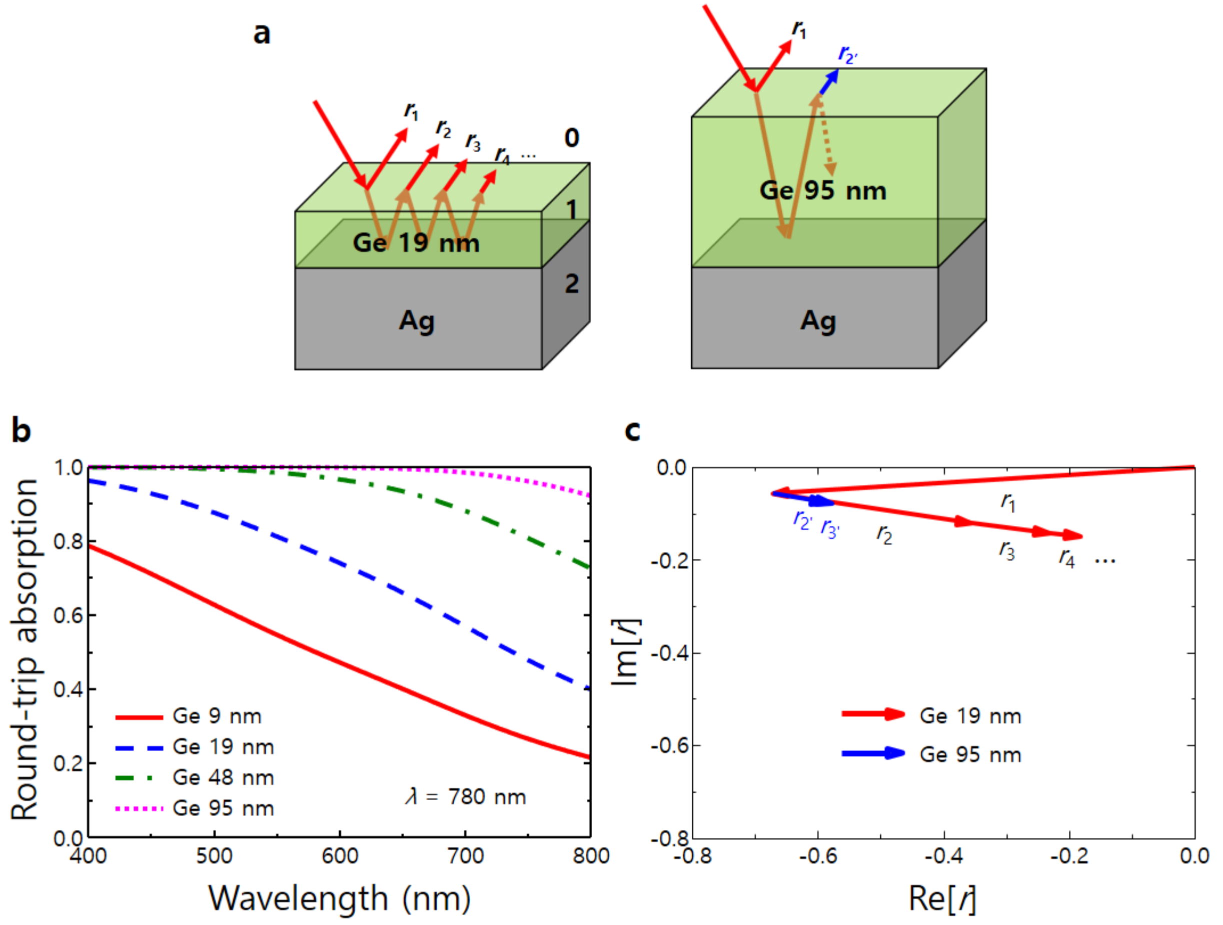

Figure S2. Interference properties of the two-layer optical absorbers. (a) Schematic diagrams of an interference for the Ge 19 nm/Ag and Ge 95 nm/Ag cases. (b) One round-trip absorption in the Ge layer with different Ge thickness at the wavelength of 780 nm. (c) Trajectory of partially reflected waves of the Ge 19 nm/Ag (red arrow) and Ge 95 nm/Ag (blue arrow) cases (at 780 nm).

The amount of absorption of the two-layer optical absorbers is not proportional to the thickness of the Ge layer. To investigate, we compared the Ge 19 nm/Ag and Ge 95 nm/Ag cases. Figure S2a shows schematic diagrams of a destructive interference of each case. To make zero reflection, the first partially reflected wave (r_1) should destructively interferes with the other waves (r_2, r_3…). In Figure S2b, the absorption spectra from the Ge layer (9, 19, 48, and 95 nm) within one round-trip at the wavelength of 780 nm are shown. For the Ge 9, 19 nm cases, destructive or constructive interference between r_1 and other reflected waves (r_2, r_3…) can be occurred because much portion of the light is left even after a round-trip. However, when the Ge layer is as thick as 95 nm (magenta dotted line), there is little light after a round-trip, which cannot induce any interference. It can be

intuitively understood by showing the trajectory of partially reflected waves in complex plane. The partially reflected waves can be obtained by

$$r = \sum_{m=0}^{\infty} r_{m+1} = r_1 + \sum_{m=1}^{\infty} t_{01} t_{10} {r_{10}}^{m-1} {r_{12}}^{m} e^{i2m\beta_1} \quad (1)$$

where r is the reflection coefficient of the two-layer system, r_m is the mth partially reflected waves, $r_{mn} = (p_m - p_n)/(p_m + p_n)$, $t_{mn} = 1 + r_{mn}$, $p_m = n_m$ for normal incidence, n_m is the complex refractive indices of the mth layer, and β_1 is the longitudinal wavenumber of inside the Ge layer. The partially reflected waves calculated by Equation (1) are shown in Figure S2c. As expected, the Ge 19 nm case destructively interfere more than the Ge 95 nm case, showing lower reflection (close to the origin point).

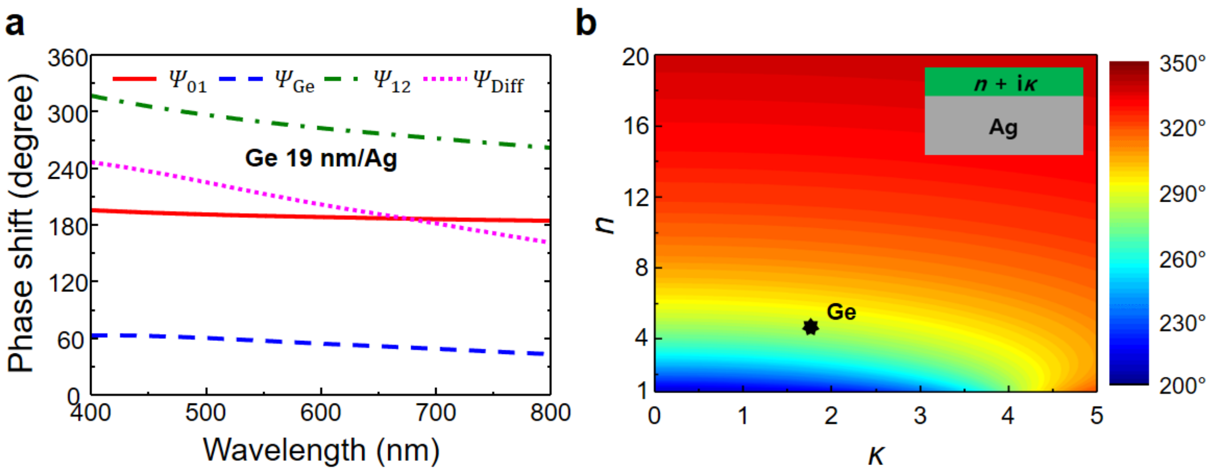

Figure S3. (a) Phase-matching condition for the Ge 19 nm/Ag case depending on the wavelength. (b) Phase shift at the Ag surface depending on the refractive index of environment at the wavelength of 600 nm. The refractive index of the Ge is marked by black hexagon.

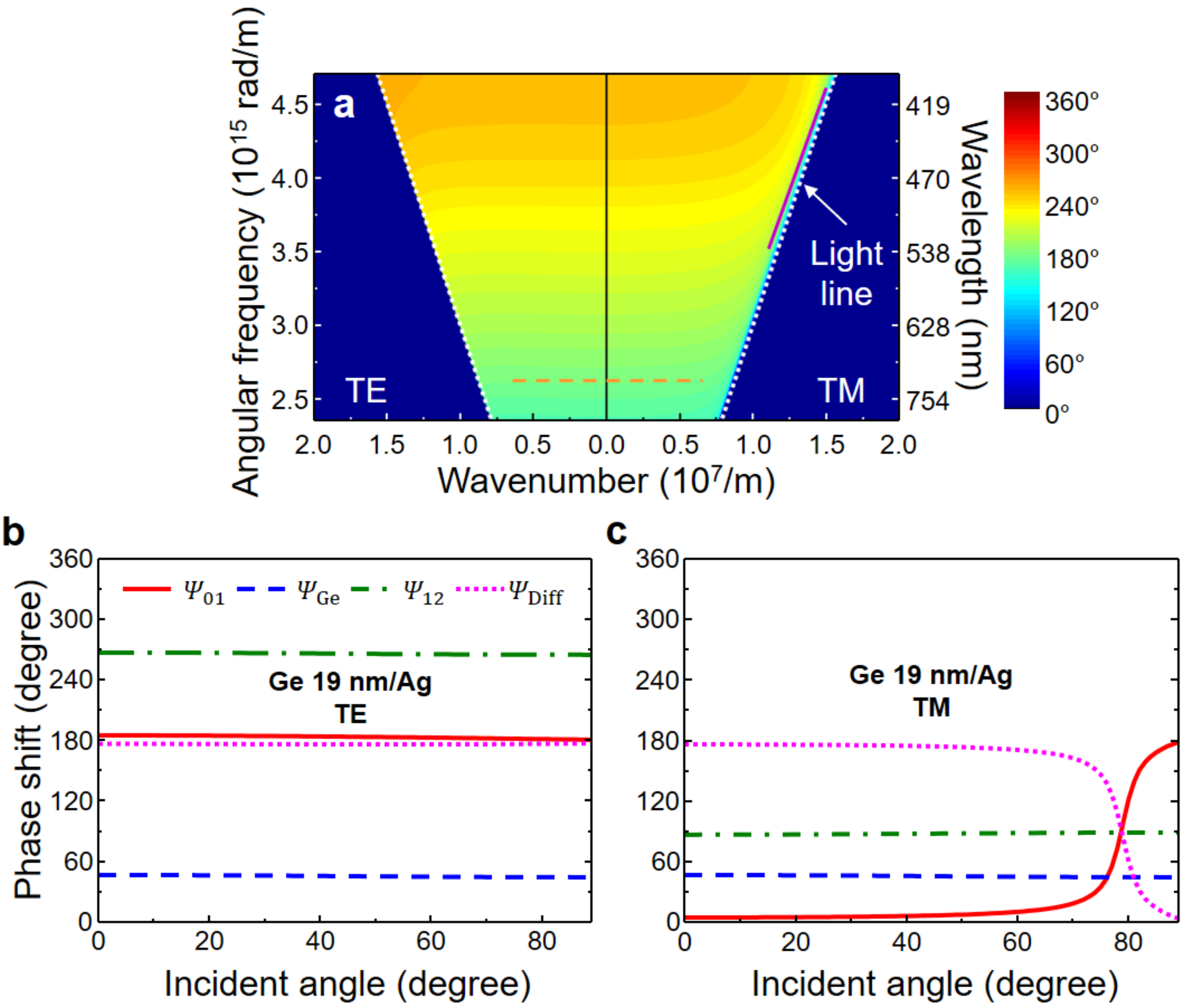

Figure S4. Dispersion relationship of (a) Phase difference (Ψ_{Diff}) for the Ge 19 nm/Ag case of TE (left panel) and TM (right panel) polarization. The white dotted lines represent the light line in air. The flat dispersion region (720 nm) is marked by orange dashed line. Phase shifts at the flat dispersion region are shown depending on the incident angle for (b) TE and (c) TM polarization. The abrupt phase change in the TM case is caused by the Brewster angle.

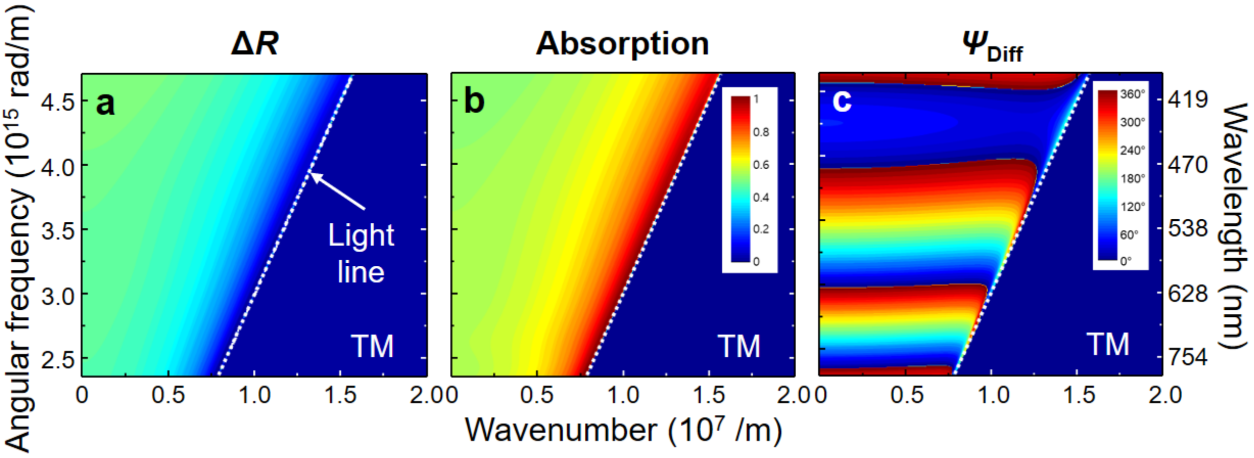

Figure S5. Dispersion relationship of (a) Magnitude difference (ΔR), (b) Absorption and (c) Phase difference (Ψ_{Diff}) for the Ge 300 nm/Ag case of TM polarization. The dotted lines represent the light line in air. The insets show scale bar (ΔR is same with the absorption).

R_{01} is always nearly zero at the Brewster angle regardless of the Ge thickness and $R_{12}\exp(-\alpha d_1)$ can also be close to zero with relatively small thickness d_1 (since α is large, $d_1 \geq 10$ nm in our case). Therefore, the magnitude matching ($\Delta R = 0$) and the high slope region *almost* always exists regardless of the Ge layer thickness. To prove it, we show the dispersion relationship of the magnitude difference (ΔR), the absorption and the phase difference (Ψ_{Diff}) for the Ge 300 nm/Ag case in Figure S5 (a-c) (only for the TM polarization). The horizontal axis represents the surface-parallel component of the wavevector, β (m^{-1}), and the vertical axis is the angular frequency, ω (rad/m), corresponding to the visible wavelength. The dotted lines represent the light lines in air. The absorption follows ΔR rather than Ψ_{Diff}. The magnitude-matching ($\Delta R = 0$) is occurred at the Brewster angle, and the near unity absorption is also shown there, while the phase-matching condition ($\Psi_{\mathrm{Diff}} = \pi$) is not satisfied. This indicates that the supported Brewster mode in this region is a non-radiative mode, which is almost no reflected light in air (Ψ_{Diff} is not important), and this mode is independent on the thickness of the Ge layer. This region corresponds to the high slope region in the main text, and there is no flat dispersion region due to the thick Ge layer. Moreover, this non-radiative mode of the two-layer system is also present in other lossy semiconductors, where absorption coefficients are not zero and thicknesses are enough to make $R_{12}\exp(-\alpha d_1) \simeq 0$. This mode also appears whether the metal back reflector exists or not, if the absorbers can sufficiently absorb incident light ($\exp(-2\alpha d_1) \simeq 0$).

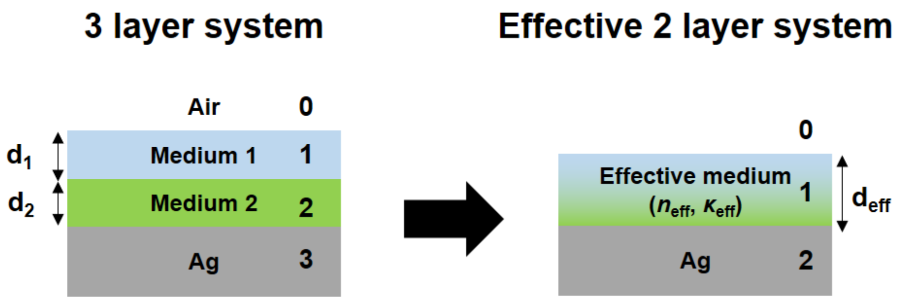

Figure S6. Schematic of the three-layer and the effective two-layer system.

The reflection coefficient of the three-layer system can be expressed as:

$$r_{0123} = \frac{r_{01} + r_{123}e^{-2i\beta_1 d_1}}{1 + r_{01}r_{123}e^{-2i\beta_1 d_1}} \quad (1)$$

$$r_{123} = \frac{r_{12} + r_{23}e^{-2i\beta_2 d_2}}{1 + r_{12}r_{23}e^{-2i\beta_2 d_2}} \quad (2)$$

where $r_{mn} = (p_m - p_n)/(p_m + p_n)$ is the reflection coefficient at the interface between the mth and n^{th} layer, and $p_m = n_m\cos(\theta_m)$ for TE polarization and $p_m = n_m/\cos(\theta_m)$ for TM polarization. Here, n_m is the complex refractive index of the mth layer, the propagation angle $\theta_m = \sin^{-1}(\sin(\theta_0)/n_m)$ is obtained by Snell's law, θ_0 is the incident-angle from the air ($m = 0$), β_m is the longitudinal wavenumber inside the mth layer, and d_m is the thickness of the mth layer. The reflection coefficient of the two-layer system can also be expressed as:

$$r_{012} = \frac{r_{01} + r_{12}e^{-2i\beta_1 d_1}}{1 + r_{01}r_{12}e^{-2i\beta_1 d_1}} \quad (3)$$

For the effective two-layer system, the reflection coefficients are given by:

$$r_{01} = \frac{\cos(\theta_0) - n_{eff}\cos(\theta_1)}{\cos(\theta_0) + n_{eff}\cos(\theta_1)} \quad (4)$$

$$r_{12} = \frac{n_{eff}\cos(\theta_1) - n_2\cos(\theta_2)}{n_{eff}\cos(\theta_1) + n_2\cos(\theta_2)} \quad (5)$$

The total reflection coefficient of the effective two-layer system can be calculated by inserting

Equation (4), (5) into Equation (3). The three-layer system can be understood as the effective two-layer system as shown in Figure S6. Therefore, the effective refractive index is obtained by numerically solving $r_{0123} = r_{012}$ (Equation 1 equals to Equation 3).

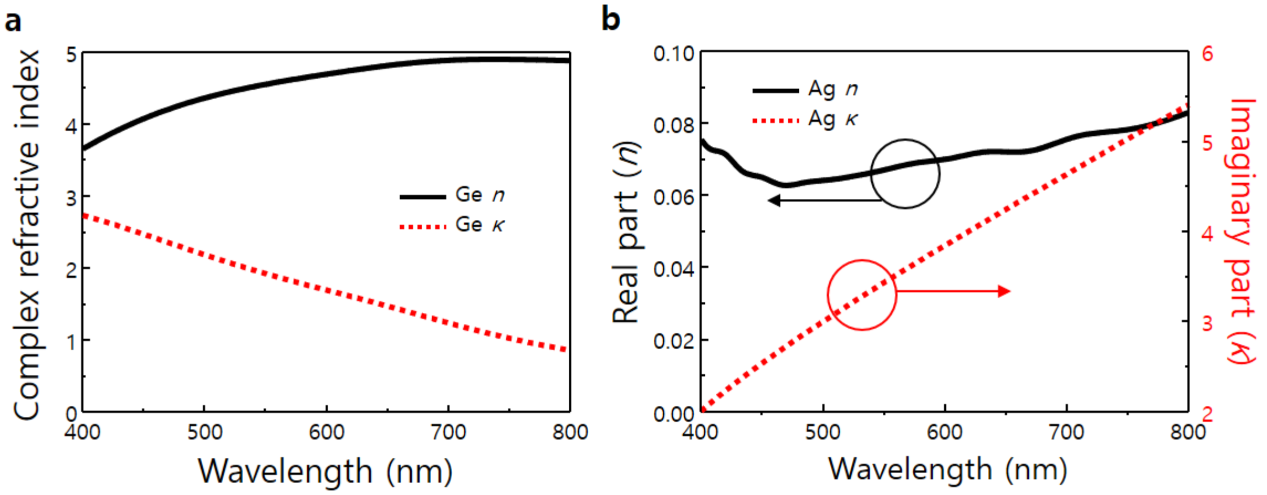

Figure S7. Complex refractive indices of a) Ge and b) Ag measured by an ellipsometer. The black solid line and the red dotted line represent the real and imaginary parts of the refractive indices.

Supplementary information references

1 McPeak, K. M. *et al.* Plasmonic films can easily be better: Rules and Recipes. *ACS Photonics*, **2**, 326-333 (2015).
